# A Review of Stage 0 Biomarkers in Type 1 Diabetes: The Holy Grail of Early Detection and Prevention?

**DOI:** 10.3390/jpm14080878

**Published:** 2024-08-20

**Authors:** Măriuca Mănescu, Ion Bogdan Mănescu, Alina Grama

**Affiliations:** 1 Department of Pediatrics, Emergency County Clinical Hospital of Targu Mures, 50 Gheorghe Marinescu, 540136 Targu Mures, Romania; alina.grama@umfst.ro; 2 Department of Laboratory Medicine, Faculty of Medicine, George Emil Palade University of Medicine, Pharmacy, Science, and Technology of Targu Mures, 38 Gheorghe Marinescu, 540142 Targu Mures, Romania; bogdan.manescu@umfst.ro; 3 Department of Pediatrics, Faculty of Medicine, George Emil Palade University of Medicine, Pharmacy, Science, and Technology of Targu Mures, 38 Gheorghe Marinescu, 540142 Targu Mures, Romania

**Keywords:** at-risk children, at-risk infants, biomarkers, early detection, early prediction, islet autoimmunity, seroconversion, stage 0, T1D, type 1 diabetes mellitus

## Abstract

Type 1 diabetes mellitus (T1D) is an incurable autoimmune disease characterized by the destruction of pancreatic islet cells, resulting in lifelong dependency on insulin treatment. There is an abundance of review articles addressing the prediction of T1D; however, most focus on the presymptomatic phases, specifically stages 1 and 2. These stages occur after seroconversion, where therapeutic interventions primarily aim to delay the onset of T1D rather than prevent it. This raises a critical question: what happens before stage 1 in individuals who will eventually develop T1D? Is there a “stage 0” of the disease, and if so, how can we detect it to increase our chances of truly preventing T1D? In pursuit of answers to these questions, this narrative review aimed to highlight recent research in the field of early detection and prediction of T1D, specifically focusing on biomarkers that can predict T1D before the onset of islet autoimmunity. Here, we have compiled influential research from the fields of epigenetics, omics, and microbiota. These studies have identified candidate biomarkers capable of predicting seroconversion from very early stages to several months prior, suggesting that the prophylactic window begins at birth. As the therapeutic landscape evolves from treatment to delay, and ideally from delay to prevention, it is crucial to both identify and validate such “stage 0” biomarkers predictive of islet autoimmunity. In the era of precision medicine, this knowledge will enable early intervention with the potential for delaying, modifying, or completely preventing autoimmunity and T1D in at-risk children.

## 1. Introduction

Type 1 diabetes mellitus (T1D) is an incurable autoimmune disease characterized by the destruction of pancreatic islet cells, resulting in lifelong dependency on insulin treatment. This disease has a significant genetic component, with up to 50% of the risk factors being hereditary [1]. Populational studies indicate that the risk of T1D occurrence is higher among relatives of T1D patients, and the concordance rate in monozygotic twins ranges from 25% to 50%, underscoring a substantial genetic influence [2,3,4]. The association between T1D and the human leukocyte antigen (HLA) complex was first reported in 1973, following the discovery that certain HLA antigens were more prevalent in T1D patients [5]. Genetic investigations, including linkage analysis and genome-wide association studies, have been conducted to support these observations [6,7,8,9,10,11,12,13,14,15,16]. Subsequent studies have confirmed that *HLA-DR* and *HLA-DQ* genes exhibit the strongest association with T1D, with certain haplotypes conferring a high risk (e.g., *HLA DR4-DQ8* or *DR3-DQ2*) and others providing protection (e.g., DR2) [17,18].

Given these genetic insights and the familial aggregation of T1D, it is possible to identify individuals at risk, ideally through the screening of newborns. Consequently, a primary goal in T1D management has always been early detection and prevention rather than treatment. While various biomarkers have been associated with T1D, most of the currently studied and validated biomarkers only predict T1D after the pathophysiological process has begun. For instance, the presence of multiple autoantibodies is a well-known predictor of T1D progression. Other biomarkers, such as dysglycemia and glycated hemoglobin, also serve as late indicators of the disease. To effectively prevent T1D, it is crucial to develop accurate predictive tools that can identify high-risk individuals before the onset of the pathophysiological process and occurrence of islet autoimmunity (IA). Currently, the development and application of such predictive tools are primarily within the research domain rather than clinical practice. However, the growing interest in early detection and prevention of T1D, along with recent technological and therapeutic advancements, underscore the need for increased focus and investment in this area.

There is an abundance of review articles addressing the prediction of T1D; however, most focus on the presymptomatic phases, specifically stages 1 and 2. These stages occur after the onset of IA, where therapeutic interventions primarily aim to delay the onset of T1D rather than prevent it. This raises a critical question: what happens before stage 1 in individuals who will eventually develop T1D? Is there a “stage 0” of the disease, and if so, how can we detect it to increase our chances of truly preventing T1D? In pursuit of answers to these questions, this narrative review aims to highlight recent research in the field of early detection and prediction of T1D, specifically focusing on biomarkers that can predict T1D before the onset of IA.

## 2. Past and Ongoing Efforts in the Study of T1D

Due to the interplay of genetic and environmental risk factors, as well as its profound impact on patients’ lives, often starting at a very young age, T1D has been a focal point of scientific research. This includes efforts to identify new genes associated with T1D risk, beyond HLA, and to explore maternal, environmental, and dietary factors. Several large cohort studies have undertaken the challenge of investigating the risk factors, triggers, and development of T1D. Although the primary goals of these studies were not specifically aimed at discovering “stage 0” biomarkers, they have nevertheless provided valuable biological samples that have been instrumental in many of the studies cited in this review. The advantage of these cohort studies lies in their longitudinal design, where biological samples are collected and stored at birth, or shortly after, and followed up at regular intervals until the development of T1D or a specified age, depending on the study. Thus, before delving into the study of “stage 0” biomarkers, some of these large initiatives that have contributed significantly to our understanding of T1D or are still ongoing, are presented in Table 1 [19,20,21,22,23,24,25,26,27,28,29,30,31,32,33].

The studies referenced in this review are systematically categorized by their respective research domains, including epigenetics, transcriptomics, proteomics, metabolomics, lipidomics, and gut microbiota. Each study is described in the main text, with a summary of all discussed biomarkers provided in Table 2.

## 3. Epigenetics

A longitudinal analysis by Johnson et al. involving 87 case–control pairs from the DAISY cohort revealed significant age-related differences in peripheral whole blood DNA methylation across 10 genomic regions. Some methylation differences were detectable as early as birth and persisted through and after the onset of islet IA. These alterations were associated with transcription factors, protein-coding genes, and non-coding regions with potential regulatory roles, suggesting that early epigenetic changes may be linked to T1D. However, further validation is necessary to confirm these findings [34].

A small study (n = 7 matched case–control pairs) by Starskaia et al. identified early DNA methylation changes in individuals predisposed to T1D, before diagnosis and even before the onset of IA. The researchers analyzed fractionated PBMC samples, enabling the detection of immune cell subset-specific DNA methylation alterations. Significant methylation changes were found in CD4+ T cells, CD8+ T cells, and CD4− CD8− cell fractions. Notably, methylation changes were observed in genes previously associated with T1D, such as *IL32*, *TRAF3*, and *DGKQ*, as well as in novel candidate genes not previously linked to the disease, including *ARRDC2* and *PCBP3*. These findings underscore the potential of DNA methylation as an early biomarker for T1D and highlight the importance of immune cell-specific analyses in understanding the disease’s pathogenesis [35].

## 4. Transcriptomics

### 4.1. Specific Immune Cell-Related Transcriptome Signatures Predict T1D

Mehdi et al. examined peripheral blood longitudinal microarray data from 92 children enrolled in the BABYDIET and DIPP cohorts to identify gene expression signatures linked to T1D risk. The research revealed distinct gene expression patterns associated with seroconversion within the first year of life, identifying 67 differentially expressed genes related to T cell, dendritic cell, and B cell responses. HLA scores together with near-birth expression levels of *ADCY9*, *PTCH1*, *MEX3B*, *IL15RA*, *ZNF714*, *TENM1*, and *PLEKHA5*, were able to predict seroconversion with an AUC of 0.850. The study highlighted the involvement of the ubiquitin–proteasome pathway in T1D susceptibility and islet autoimmunity development. Researchers proposed that early gene expression profiling could predict the risk of seroconversion and inform therapeutic interventions [36].

A smaller study by Kallionpää et al. investigated early molecular markers in seven children who developed IA and matched controls. Using RNA sequencing, they analyzed unfractionated peripheral blood mononuclear cells, as well as CD4+ T cells, CD8+ T cells, and CD4− CD8− fractions. The research identified several upregulated transcripts, including interleukin 32 (IL-32), prior to IA. Single-cell RNA sequencing revealed that the elevated IL-32 levels primarily originated from activated T cells and natural killer cells. Additionally, the study demonstrated that IL-32 expression in pancreatic islets and beta cells can be induced by viruses and cytokines. This upregulation of IL-32 before seroconversion underscores its significant role in the immunological profile of children at risk for developing β-cell autoimmunity and suggests it may serve as a predictive biomarker for IA [37].

A study by Xhonneux et al. analyzed longitudinal blood transcriptomes from 2013 samples of 400 individuals in the TEDDY study to identify gene expression changes before both T1D and islet autoimmunity. It found significant age-associated gene expression changes in healthy infants and distinct, age-independent changes linked to disease progression. Notably, unique transcriptional signatures were associated with varying progression rates based on the initial islet autoantibody type (IAA or GADA). A specific NK cell-based signature was identified, showing increased expression with progression toward both islet autoimmunity and T1D, and this finding was later validated in the independent DIPP study. This NK cell signature suggests a potential role in T1D pathogenesis. The exact role of NK cells in insulitis remains uncertain, with further investigation needed to determine if their involvement is a direct cause of T1D, a response to infection, or both. The study also developed a predictive model that integrated gene expression and autoantibody data to estimate individual T1D risk, demonstrating strong predictive performance and providing a foundation for early monitoring and intervention strategies for at-risk infants [38].

Lin et al. analyzed longitudinal whole blood transcriptome sequencing in 418 case–control pairs from the TEDDY study to investigate immune responses for up to 12 months before developing IA. The study revealed that children who first developed insulin-specific autoantibodies exhibit distinct transcriptional profiles compared to those who developed GADA autoantibodies first, with *GSTM1* gene dosage linked to GADA positivity. Additionally, 9–12 months before IA, there was an increase in monocytes and a decrease in B cell proportions, especially in those developing insulin antibodies first. While control children showed strong immune responses to enterovirus infection, the response was weaker in those who later developed IA. These results underscore significant immune-related transcriptomic differences between children who progress to IA and controls, highlighting a deficiency in antiviral responses in at-risk children. Moreover, these changes may be used as early biomarkers to predict the occurrence of IA [39].

Overall, the onset of IA and T1D appears to be preceded by changes in transcriptomic signatures across various immune cell types. These include cells of the innate immune system, such as dendritic cells and NK cells, as well as those of the adaptive immune system, such as T cells and potentially B cells. This highlights the complexity and multitude of factors involved, illustrating the intricate pathways that become dysregulated before the onset of autoimmunity.

### 4.2. Upregulation of Type I Interferon Transcriptomic Pathways Precedes T1D

In another study by Kallionpää et al., the authors investigated early biomarkers of T1D by analyzing transcriptomic data from at-risk individuals. Using a comprehensive approach, they examined whole-blood RNA samples from a cohort of 28 children, including nine individuals in the pre-seroconversion stage. The study identified significant early activation of type 1 interferon (IFN) response pathways, detectable even before the appearance of autoantibodies. The research indicated that this early IFN transcriptional signature, characterized by the upregulation of numerous genes linked to innate immune responses, could serve as a potential biomarker for predicting disease onset, providing valuable insights into the timing and mechanisms of immune system activation in T1D [40].

Ferreira et al. explored the potential of the type 1 IFN gene signature as an early biomarker for T1D. Using DNA microarray analysis, they assessed the IFN-inducible transcriptional signature in PBMCs across various groups, including 49 patients with recently diagnosed T1D, 15 patients with long-standing T1D, 93 healthy adult volunteers, and 109 genetically predisposed children from the BABYDIET study, a prospective birth cohort study. Similarly to Kallionpää et al., the researchers analyzed a predefined set of 225 IFN signature genes and found significantly elevated IFN signature expression in genetically predisposed children before the development of autoantibodies (*p* = 0.0012), but not in individuals with established T1D. This elevated expression was transient, correlating with recent upper respiratory infections and increased expression of CD169, a lectin-like receptor on monocytes [41].

Given the known role of IFN in antiviral protection, these concurrent findings of Kallionpää et al. and Ferreira et al. support the long-standing belief that viral infections are major environmental triggers for T1D and propose that IFN upregulation could be a key mechanistic link in this process, as well as an early biomarker of IA and T1D.

## 5. Proteomics

### 5.1. Changes in Apolipoproteins and Complement Proteins Precede the Onset of Autoimmunity

Moulder et al. published a paper exploring the use of proteomics to predict which children, identified at birth as at risk for T1D based on the HLA genotype, would remain autoantibody-negative and which would develop autoantibodies and eventually T1D. All children studied were participants in the Finnish DIPP study, with prospective sampling performed at each visit between 3 months and 12 years of age. The proteomic analysis was conducted on 19 children who eventually developed T1D and 19 matched controls, with 13 case–control pairs processed for iTRAQ analysis and six pairs analyzed using a label-free approach. A comparison between the methods revealed an overlap of a core of 248 proteins. Throughout the study, distinct differences were observed between children who developed T1D and their controls: lower levels of apolipoprotein C-II (APOC2), apolipoprotein C-IV (APOC4), and mannose-binding protein C (MBL2); and higher levels of complement factor H-related protein 5 (FHR-5) and complement component C9 (CO9). However, when only pre-seroconversion samples were compared, the differences were limited to lower levels of APOC4 (throughout the entire period, and specifically 9–12 months and 3–6 months before seroconversion), lower levels of APOC2 (throughout the entire period), and higher levels of profilin-1 (PFN1, only 3–6 months before seroconversion). Case- and control-to-reference ratios indicated longitudinal changes in the serum proteome. Out of the 26 proteins (correlation coefficient > 0.4), 14 were increased and 12 were decreased. The most frequently observed enriched functional annotations for protein profiles distinct between children progressing to T1D and their controls included lipid and cholesterol transport, acute inflammatory response, and humoral and innate immunity, with a notable representation of complement proteins. A top scoring pairs (TSP) analysis achieved a classification accuracy of 91% (AUC 0.850) for distinguishing children progressing to T1D. This classification relied on the relative levels of APOC4, which were lower, and afamin (AFAM), which were higher in those progressing compared to the controls. Additionally, when examining longitudinal changes in subjects progressing to T1D, TSP analysis distinguished pre- and post-seroconversion samples with an accuracy of approximately 80%. This differentiation was based on the changes in abundance of apolipoprotein A-IV (APOA4) and insulin-like growth factor-binding protein complex acid labile subunits [42].

Webb-Robertson et al. examined 172 children from the DAISY study, with multiple plasma samples collected over time. The cohort included 40 controls and 132 cases, although only 47 cases had samples collected prior to IA occurrence due to inconsistent sampling. Control children were matched based on frequency, age, sex, and HLA genotype. Complement proteins were measured using two methods: selected reaction monitoring-based targeted proteomics and a CLIA-validated immunoassay. The analysis revealed a consistent pattern of decreased complement proteins, except for MBL2, prior to the appearance of IA. The classical and lectin pathways exhibited the strongest decrease in complement proteins, as observed through both statistical and machine learning analyses. The proteins most significantly associated with the development of IA were C3a, C1r, C1s, C3b, and C3. Conversely, elevated levels of MBL2 were linked to IA occurrence. The authors concluded that panels of several complement proteins would be effective as predictive tools. Decreased levels of multiple complement proteins present a promising biomarker candidate for the prediction of T1D and may be involved in disease development. Notably, these findings appear to contradict the earlier results of Moulder et al. Although the two studies did not examine identical complement proteins, both included MBL2 in their analyses. Moulder et al. found that the occurrence of IA was associated with lower levels of MBL2 and higher levels of certain complement proteins. In contrast, Webb-Robertson et al. reported that IA occurrence was linked with higher levels of MBL2 and lower levels of many complement proteins [43].

### 5.2. Extracellular Matrix and Innate Immunity Are Regulated en Route to T1D

In a blinded, two-phase case–control analysis of the TEDDY study, samples were collected at various time points up to 6 years of age to establish the moment of seroconversion, if applicable, and to analyze the proteome. The study aimed to utilize proteomics to identify biomarkers predictive of T1D development. The study concluded when participants reached the age of six and the participants were divided into three groups based on their outcome: control (healthy), islet autoimmunity (still normoglycemic), and T1D. An untargeted proteomics analysis identified 376 proteins involved in pathways such as complement and coagulation cascades, extracellular matrix interactions, antigen presentation, inflammatory signaling, nutrient absorption and digestion, and cellular metabolism. Further validation of these biomarkers resulted in 83 biomarkers being confirmed in a group of 990 individuals for both IA and T1D. Through machine learning analysis, panels of proteins were identified that could predict both the presence of persistent autoantibodies with normoglycemia (AUC 0.871) and the onset of T1D (AUC 0.918) by the age of six. Most notably, the proteome changes necessary for this prediction occurred six months before seroconversion, allowing for the timely prediction of autoimmunity even before stage 1 of the disease [44].

### 5.3. Integration of Data from Disparate Sources for T1D Prediction

In a study by Frohnert et al., 67 children from the DAISY cohort, which included high-risk individuals (first-degree relatives of T1D patients or children with type 1 diabetes susceptibility, given HLA DR-DQ genotypes identified through newborn screening), were analyzed. The cohort consisted of 22 children who developed T1D, 20 who developed persistent IA and were positive for autoantibodies at their last study visit, and 25 control subjects. The study examined samples from the earliest visit (9–15 months) and samples just before the onset of autoimmunity, analyzing the relative abundance of 1001 serum proteins, a cytokine panel, and 106 non-HLA relevant SNPs. These data, along with metadata (age at sampling, HLA risk group, sex, ethnicity, and family history of T1D), were incorporated into a machine learning model for the prediction of IA. The prediction was not associated with the absolute values of the investigated markers, but with changes in these values between the two timepoints. Among the top features selected by the algorithm were all five metadata elements. From the SNPs investigated, *PTPN22* (rs2476601) and *CTLA4* (rs3087243 and rs231775) were identified as markers associated with the development of autoimmunity. Many of the top features selected were metabolites, with ascorbate (vitamin C) being the most prominent. At the earliest timepoint, ascorbate showed a lower relative abundance in participants who developed autoimmunity compared to controls, with these levels increasing over time, while controls showed a decrease over time. These differing trajectories of ascorbate were significantly associated with IA outcomes. Other metabolites predictive of IA included 3-methyl-oxobutyrate, 4-hydroxyhippuric acid, and pyroglutamic acid. In terms of proteins, the algorithm frequently selected Fc receptor-like protein 3 (FCRL3), killer cell lectin-like receptor K1 (KLRK1), matrix metalloproteinase-2 (MMP-2), activin A, structure-specific recognition protein 1 (SSRP1), and casein kinase II subunit alpha. Based on these biomarkers, the algorithm predicted the occurrence of IA with a high accuracy (AUC 0.910). This study not only identified several novel biomarkers but also innovatively showed how integrating data from disparate sources such as metadata, metabolomics, proteomics, and genetics into a single algorithm could prove to be an effective way of predicting the occurrence of IA [45].

## 6. Metabolomics and Lipidomics

### 6.1. Lower Triglycerides and Phospholipid Levels at Birth Predict T1D

La Torre et al. analyzed cord blood lipids in 76 children who developed T1D by the age of eight and 76 controls matched for the mother’s age, gestational age, date of birth, sex, and HLA genotype. Their study revealed lower levels of phosphatidylcholines and phosphatidylethanolamines in the cord blood of T1D patients diagnosed before the age of 8 years old. Also, lower levels of triglycerides were observed, but this finding was influenced by gestational age [46].

In a study including 33 T1D patients and similar groups of children who developed three or four, two, or one islet autoantibody, Orešič et al. reported that T1D progressors can be predicted by lower levels of choline-containing phospholipids, including sphingomyelins and phosphatidylcholines. The researchers proposed a molecular signature based on seven lipids that was able to predict a significantly higher risk of progression to T1D (odds ratio of 5.94, 95% confidence interval 1.07–17.50) [47].

In a study by Lamichhane et al., cord blood lipids were analyzed in 30 T1D progressors, 33 IA (but not T1D) progressors, and 38 age-matched controls. The results revealed lower levels of phospholipids, specifically sphingomyelins, and higher levels of cholesterol esters in T1D progressors. The researchers proposed a molecular signature based on five lipids that showed high predictive performance for progression to T1D (AUC 0.830) [48].

### 6.2. Lower Triglyceride and Phospholipid Levels Persist en Route to IA and T1D

In an older longitudinal prospective study also performed by Orešič et al., serum metabolome changes were analyzed in 56 children who progressed to T1D and 73 healthy controls. The results revealed that, at birth, levels of succinic acid and phosphatidylcholine were lower in children who later developed T1D. Throughout the follow-up period, these children also exhibited lower levels of triglycerides, antioxidant ether phospholipids, and sphingomyelins. In the months preceding autoimmunity, seroconversion was marked by elevated levels of proinflammatory lysophosphatidylcholine and glutamic acid, alongside decreased levels of ketoleucine. These changes in lipid metabolites were found to be independent of HLA-associated genetic risk. Interestingly, the metabolomic alterations partially recovered after seroconversion [49].

In a similar manner, another prospective study by Lamichhane et al. compared lipidomic signatures of 40 children who developed T1D, 40 who developed IA, and 40 matched controls. Similar to Orešič et al., the researchers found that sphingomyelins were persistently decreased in T1D progressors. Also, the T1D group exhibited lower phosphatidylcholine and triglycerides at the age of three months [50].

In a longitudinal study by Sen et al., which included 34 T1D progressors, 27 IA (but not T1D) progressors, and 10 controls matched for HLA profile, age, and sex, it was found that levels of most lipids and polar metabolites were lower in PBMCs of T1D or IA progressors compared with controls. Metabolic processes linked to alanine, glutamate, aspartate, glycerophospholipids, and sphingolipids were over-represented in PBMCs. Additionally, specific alterations in ceramide pathways were associated with the progression to T1D [51].

## 7. Intestinal Microbiota

The TEDDY study analyzed 10,913 metagenomes collected longitudinally from 783 children, including 415 controls, 267 seroconverters, and 101 diagnosed with T1D, approximately monthly during their first five years of life. Participants were recruited from six clinical centers across Finland, Sweden, Germany, Washington, Georgia, and Colorado. The researchers found that, compared with healthy controls, IA progressors had lower levels of *Lactobacillus rhamnosus* and *Bifidobacterium dentium*, along with a higher abundance of *Streptococcus* group *mitis*/*oralis*/*pneumoniae* species. Moreover, compared with controls, T1D progressors showed lower levels of *Streptococcus thermophilus* and *Lactococcus lactis* along with higher levels of *Bifidobacterium pseudocatenulatum*, *Roseburia hominis*, and *Alistipes shahii* [52].

In a cohort study by Davis-Richardson et al., which involved 76 children with a high genetic risk for T1D monitored from birth to 2 years of age, metagenomic analyses revealed significant microbiome alterations prior to the onset of IA. Specifically, *Bacteroides dorei* and *Bacteroides vulgatus* were found to be significantly more abundant in cases compared to controls before seroconversion. The abundance of *Bacteroides dorei* peaked at 7.6 months in cases, which was more than 8 months before the occurrence of IA. This suggests that early microbiome changes, particularly the increased presence of *Bacteroides dorei*, could serve as potential predictors of T1D autoimmunity in genetically susceptible infants [53].

However, a study by Kostic et al. suggests that significant changes in gut microbiota occur subsequent to seroconversion. The Finnish longitudinal study involving 33 infants at risk for T1D, monitored from birth through 3 years of age, investigated the composition of the gut microbiota in these children using 16S rDNA sequencing. Serum and stool metabolomics were also carried out. The research demonstrated that while there are shifts in taxonomic composition over time, the trajectories of these taxonomic changes remain remarkably consistent throughout infancy. Notably, a 25% reduction in alpha diversity was observed after seroconversion in those who later developed T1D. This reduction, specific to individuals who progress to T1D, is not seen in seroconverters who do not advance to disease. The observed microbial shift occurs after seroconversion but precedes the clinical onset of T1D [54].

Extending beyond the bacterial microbiota, a small study by Zhao et al. analyzed the intestinal virome in 11 children at risk for T1D and controls in order to identify changes occurring prior to the appearance of IA. It was found that children who later developed T1D exhibited a less diverse and distinct virome compared to controls. Specifically, eukaryotic viruses, including those from the *Circoviridae* family, were more prevalent in controls, while these sequences were less common in cases. Additionally, bacteriophage diversity and richness were higher in controls. Notably, the observed reductions in *Circoviridae*-related sequences and the overall virome diversity in children who progressed to T1D occurred before the onset of islet autoantibodies. These findings suggest that alterations in the intestinal virome precede the development of autoimmunity and may contribute to the progression toward T1D. Further investigation is needed to understand the role of these virome changes in the pathogenesis of T1D [55].

## 8. Discussion

Identifying at-risk populations for T1D has historically relied on the HLA typing of risk alleles or the presence of affected family members [56]. Recent advancements in genome-wide association studies have led to the development of polygenic risk scores, which combine HLA and non-HLA genetic risks, enabling more efficient identification of individuals at risk for T1D [56]. Although many at-risk children never develop islet autoimmunity, the cost–benefit ratio of advanced testing for early biomarkers improves when focusing on the high-risk subset. Consequently, newborn screening is a crucial initial step for predicting (and potentially preventing) T1D. This approach will become even more relevant as preventive therapies for T1D emerge. Recently, the FDA approved teplizumab, a humanized monoclonal anti-CD3 antibody, that delays the progression from stage 2 to stage 3 T1D [57]. While the development of teplizumab took decades, the rapid advancements in biotechnology offer hope for further drug discoveries. Meanwhile, it is essential to continue research aimed at more accurately identifying individuals who will develop T1D before the occurrence of islet autoimmunity. This period, from birth or even intrauterine life until seroconversion, can only be designated as “stage 0” given that, according to the current classification, stage 1 already implies the presence of islet autoimmunity.

In the quest for the early prediction of T1D, researchers have sought reliable indicators to distinguish at-risk individuals who will develop islet autoimmunity/T1D from those who will not seroconvert despite being at risk as well. This narrative review compiles influential studies that report on potential biomarkers of “stage 0”. We reviewed the literature across various fields, namely epigenetics, transcriptomics, proteomics, metabolomics, lipidomics, and intestinal microbiota. Despite the heterogeneity of these studies and their reliance on small cohorts, they all report significant differences between progressors and non-progressors even before the onset of autoimmunity. Studies in lipidomics are particularly noteworthy, as several independent research groups have consistently found decreased levels of phospholipids at birth, persisting throughout life until seroconversion. Additionally, the type I interferon pathway appears promising, with multiple research groups reporting its activation prior to seroconversion.

In conclusion, no established biomarker or panel of biomarkers for predicting the onset of autoimmunity exists to date. However, we should remain optimistic. Recent research has identified candidate biomarkers for the early prediction of T1D, before the onset of autoimmunity. As the therapeutic landscape evolves from treatment to delay, and ideally from delay to prevention, it is crucial to both identify and validate such “stage 0” biomarkers predictive of islet autoimmunity. This knowledge will enable early intervention with the potential for delaying, modifying, or completely preventing T1D in at-risk children.

## Figures and Tables

**Table 1:** Past and ongoing initiatives in the study of T1D.

Study	Eligibility Criteria	Country	Cohort Size	Study Period	Primary Endpoint	Objective	Outcome
BABYDIET [19]	Infants at risk (HLA and Family)	Germany	150	2000–2006	SC/T1D	The impact of early nutritional interventions on the autoimmune process	Delaying gluten exposure until 12 months does not significantly reduce IA risk
FINDIA [20]	Infants at risk (HLA and/or Family)	Finland	1113	2002–2005	SC		Bovine insulin-free formula reduced IA risk
TRIGR Pilot Study [21]	Infants at risk (HLA and Family)	Finland	230	2004–2008	SC/T1D		Casein hydrolysate formula reduced IA risk
TRIGR [22]	Infants at risk (HLA and Family)	Several	2159	2002–2017	SC/T1D		Casein hydrolysate formula did not significantly impact T1D risk or progression.
Pre-POInT Pilot Study [23]	Children at risk (HLA and Family)	Several	25	2009–2013	Immune response to insulin	The impact of oral insulin therapy on the autoimmune process	Administration of insulin resulted in an immune response without hypoglycemia.
Pre-POInT Early Trial [24]	Children at risk (HLA and Family)	Germany	44	2015–2017			Administration of insulin did not produce the predefined immune response outlined as the primary trial outcome.
GPPAD- POInT [25]	Infants at risk (HLA and Family)	Several	1050	2018–2024	SC/T1D		Study results not yet published.
PINIT Study [26]	Children at risk (HLA and Family)	Germany	38	2018–2021	Immune response to insulin	The impact of nasal insulin therapy on the autoimmune process	Study results not yet published.
GPPAD-SINT1A Study [27]	Infants at risk (genetic risk score or HLA/family risk)	Several	1144	2021—ongoing	SC	The impact of *B. infantis* administration on the autoimmune process	-
DAISY [28]	Risk (HLA and/or Family)	USA	2644	1993—ongoing	SC/T1D/ATA	Identification of infectious agents, dietary factors, and environmental exposures linked to an increased risk of autoimmunity and T1D.	Preliminary findings: Vitamin D intake and 25(OH)D levels are not linked to T1D risk. Increased inflammation is linked to both IA and T1D progression. Omega-3 fatty acid intake is linked to a reduced risk of IA, but not progression to T1D.
TEDDY [29]	Infants at risk (HLA and/or Family)	USA	8677	2004—ongoing	SC/T1D		Preliminary findings: Persistent enterovirus B in stool predicts IA. Children with IA have distinct gut microbiomes. Probiotics might lower risk, but antibiotics don’t affect autoimmunity. Potential benefits of vitamins D and C, and polyunsaturated fats.
ENDIA [30]	Infants at risk (Family)	Australia	1500	2013–2019	T1D		Mucosa-associated cytokines spike around the time of IA development. Continuous glucose monitoring detects early dysglycemia in individuals with IA. Multiple other findings.
MIDIA [31]	Infants at risk (HLA)	Norway	>900	2001—ongoing	SC		Preliminary findings: Respiratory infections were associated with higher risk of islet autoimmunity. Maternal obesity was associated with higher risk of IA.
DIPP [32]	Infants at risk (HLA)	Finland	17,000	1994—ongoing	SC/T1D	Identification of risk factors linked to autoimmunity and T1D.	Preliminary findings: Differences in immune cell proportions based on the initial autoantibodies. Dietary factors influence the risk of IA/T1D. Those who seroconvert have distinct lipidomic profiles.
BABYSCREEN [33]	All infants	Finland	10,400	2018—ongoing	SC/ATA		-

Risk (HLA and/or Family): individuals with HLA-associated genetic susceptibility and/or a positive family history. Abbreviations: ATA—anti-transglutaminase antibodies, DAISY—The Diabetes Autoimmunity Study in the Young, DIPP—Type 1 Diabetes Prediction and Prevention Study, ENDIA—Environmental Determinants of Islet Autoimmunity, FINDIA—Finnish Dietary Intervention Trial for the Prevention of Type 1 Diabetes, GPPAD-POInT—Global Platform of Autoimmune Diabetes, IA—islet autoimmunity, MIDIA—Miljøfaktorer I Diabetesutvikling av Autoimmunitet (which translates to “Environmental Triggers of Type 1 Diabetes”), NIP Pilot Study—Nutritional Intervention to Prevent Type 1 Diabetes Pilot Trial, PINIT Study—Primary Intranasal Insulin Trial, POInT-Primary Oral Insulin Trial, T1D—Type 1 diabetes mellitus, TEDDY—The Environmental Determinants of Diabetes in the Young, TRIGR—Trial to Reduce IDDM in the Genetically at Risk.

**Table 2:** Summary of biomarkers discussed in this review.

Cohort(s)	Size	Biomarker(s)	Finding(s)	Ref.
DAISY	174	Whole blood DNA methylation	DNA methylation changes in 10 genomic regions.	[34]
DIABIMMUNE	14	DNA methylation in PBMC fractions	DNA methylation changes in CD3+ CD4+, CD3+ CD8+, and CD4− CD8− cells.	[35]
BABYDIET and DIPP	92	Peripheral blood transcriptome signatures	A total of 67 differentially expressed genes (T cell, B cell, dendritic cell). HLA score + near-birth expression of *ADCY9*, *PTCH1*, *MEX3B*, *IL15RA*, *ZNF714*, *TENM1*, and *PLEKHA5* can predict SC (AUC 0.850).	[36]
DIABIMMUNE	14	Transcriptome of PBMC fractions	Several upregulated genes including IL-32 originating from activated T cells and NK cells.	[37]
TEDDY	400	Peripheral blood transcriptome	A specific NK cell-based signature was associated with SC and later validated in the DIPP study as well.	[38]
	936		Immune-related transcriptomic difference: weaker antiviral response associated with SC.	[39]
DIPP	28	Whole blood RNA	Early activation of type 1 interferon pathways (numerous genes linked to innate immunity).	[40]
BABYDIET	266	PBMC DNA	Transient elevated IFN signature expressions before SC, correlated with recent upper respiratory infections and CD169.	[41]
DIPP	38	Serum proteome	Changes in apolipoproteins and complement precede IA. SC is predicted with 80% accuracy by ApoA-IV and IGFALS.	[42]
DAISY	172	Plasma proteome	Decreased complement proteins are associated with SC, except for MBL2 which is elevated.	[43]
TEDDY	990	Plasma proteome	Proteome changes occur six months before SC.	[44]
DAISY	67	Serum proteome, non-HLA SNPs, metadata	An algorithm based on several biomarkers predicted SC with high accuracy (AUC 0.910).	[45]
DiPiS	152	Cord blood lipidome	Lower levels of triglycerides and phospholipids at birth predict T1D.	[46]
DIPP	286			[47]
	101			[48]
	129	Serum metabolome	Lower levels of triglycerides and phospholipids persist *en route* to IA and T1D.	[49]
	120	Plasma lipidome		[50]
	71	PBMC lipidome		[51]
TEDDY	783	Intestinal microbiota	IA progressors showed lower abundance of *L. rhamnosus* and *B. dentium*; and higher abundance of *S. mitis/oralis/pneumoniae*.	[52]
DIPP	76		Higher abundance of *B. dorei* and *B. vulgatus* before SC.	[53]
DIABIMMUNE	33		A 25% decrease in alpha diversity after SC indicates progression to T1D	[54]
	22	Intestinal virome	Decreased abundance and diversity of *Circoviridae* and bacteriophage before SC	[55]

Abbreviations: DAISY—The Diabetes Autoimmunity Study in the Young, DiPiS—Diabetes Prediction in Skåne study, DIPP—Type 1 Diabetes Prediction and Prevention study, IA—islet autoimmunity, IGFALS—insulin-like growth factor binding protein (acid labile subunit), MBL2—mannose-binding lectin-2, SC—seroconversion, SNP—single nucleotide polymorphism, PBMC—peripheral blood mononuclear cells, TEDDY—The Environmental Determinants of Diabetes in the Young study.

## Data Availability

No new data were created or analyzed in this study. Data sharing is not applicable to this article.
